# The Photophysics and Photochemistry of Melanin‐ Like Nanomaterials Depend on Morphology and Structure

**DOI:** 10.1002/chem.202102479

**Published:** 2021-10-15

**Authors:** Alexandra Mavridi‐Printezi, Arianna Menichetti, Moreno Guernelli, Marco Montalti

**Affiliations:** ^1^ Department of Chemistry “Giacomo Ciamician” University of Bologna Via Selmi 2 40126 Bologna Italy

**Keywords:** fluorescence, melanin, nanoparticles, polydopamine, transient absorption

## Abstract

Melanin‐like nanomaterials have found application in a large variety of high economic and social impact fields as medicine, energy conversion and storage, photothermal catalysis and environmental remediation. These materials have been used mostly for their optical and electronic properties, but also for their high biocompatibility and simplicity and versatility of preparation. Beside this, their chemistry is complex and it yields structures with different molecular weight and composition ranging from oligomers, to polymers as well as nanoparticles (NP). The comprehension of the correlation of the different compositions and morphologies to the optical properties of melanin is still incomplete and challenging, even if it is fundamental also from a technological point of view. In this minireview we focus on scientific papers, mostly recent ones, that indeed examine the link between composition and structural feature and photophysical and photochemical properties proposing this approach as a general one for future research.

## Graphical abstract

**Melanin‐like materials** can be easily prepared from simple precursors upon controlled oxidation/polymerization. In the resulting system, species with different structure and morphology coexist and interact. The overall photophysical and photochemical properties depend in part on the nature of the chromophores formed but also, to a large extent, on the interchromophoric interactions as typical for supramolecular architectures.

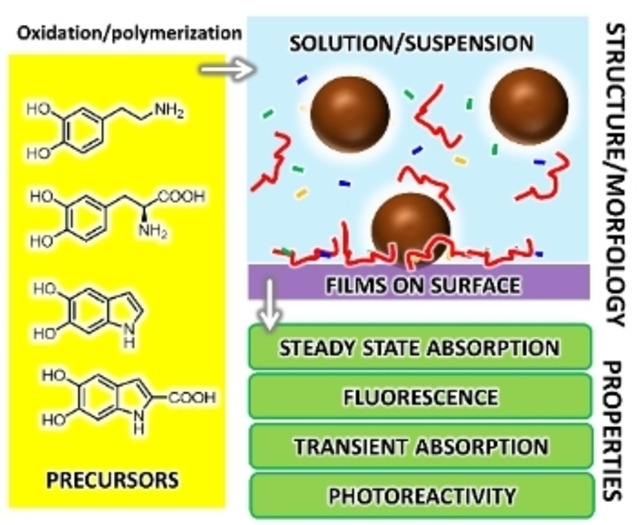

## 1 Introduction

The most important roles of melanin in nature are photo‐protection and coloration, and both involve interactions with light.^[1]^ However, activation of melanin by light may also promote harmful illnesses such as cancer.^[2]^ Therefore, the understanding of the photophysical and photochemical properties of this natural pigment is essential to unveil its biological function. Nevertheless, a complete characterization of melanin is complicated since i) the classification of the different forms of melanin present in nature (eumelanin, pheomelanin, neuromelanin, allomelanin, and pyomelanin)^[3]^ is not trivial and it is mostly based on the nature of the molecular precursors involved in their biosynthesis ; ii) it is difficult to characterize the pigment extensively in its native form and iii) it is difficult to assess if the extractive processes do not alter its nature.^[4]^

The advent of synthetic melanin‐like materials represented a real breakthrough, since these materials combine the unique optical and electronic features of natural melanin with an impressive simplicity and versatility of preparation.^[5]^ Since the preparative processes mimic, to a large extent, the proposed natural pathway,[^3b^, ^6^] in many cases synthetic melanin‐like materials have not only found a large variety of light‐based applications, but also they have become a model for the investigation of the structure of the natural pigment.^[7]^

**Figure 1:**
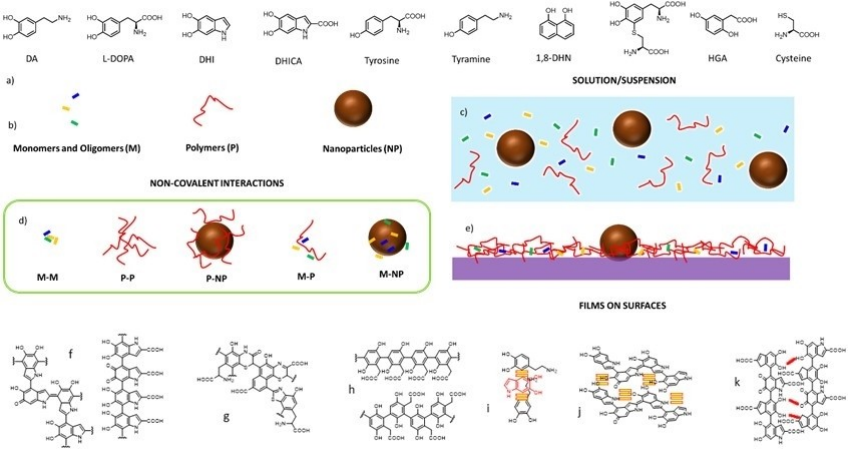
a) Precursors involved in the biosynthesis and synthesis of melanin and melanin‐like materials. b) Schematization of the different components produced during the synthesis. c) Representation of non‐interacting components in solution. d) Possible interactions between the components. e) Schematization of the composition of melanin‐based coatings. Proposed structures for f) eumelanin, g) pheomelanin, h) pyomelanin. i‐k) Proposed supramolecular interactions (π‐ π stacking, H‐bonds and H‐aromatic) between monomers and oligomers.

In this context, an impressive number of scientific papers have been published discussing the actual structure of melanin^[8]^ and in particular its polymeric or supramolecular nature,^[9]^ while on the other hand the detailed investigation of the photophysical and photochemical properties of melanin‐like materials is still incomplete.^[10]^ Even though the fascinating debate about the actual structure of melanin has still not come to an end, in this minireview we would like to discuss the correlation of the photophysical and photochemical properties of the melanin‐like materials with their variable chemical compositions and morphologies. In this perspective, it should be underlined that although the existence of several different building‐blocks in melanin‐like materials is almost generally recognized,^[11]^ it is still debated: i) what is the actual chemical composition and distribution of the different species and ii) how these components interreact and to what extend covalent and non‐covalent (supramolecular) interactions are involved in the stabilization of the structure.^[12]^ In this context, proposed structures and non‐covalent interactions are schematized in Figure 1.

As far as the morphology of melanin‐like materials is concerned, as schematized in Figure 1, they are typically produced as: i) self‐standing NP;^[13]^ ii) surface adsorbed films;^[14]^ iii) high molecular weight water soluble polymers and aggregates^[15]^ and iv) low molecular weight components. Since intercomponent interactions are known to strongly affect the photophysical and photochemical properties of the different units,^[16]^ it is believed that a connection of these features with the possible structure and morphology are essential. More in detail, in the first part of this minireview it is described briefly that very similar reactions may lead to the formation of melanin‐like materials that differ considerably in morphology and in physical and chemical properties. In the second part, the most relevant photophysical and photochemical studies done on melanin‐like materials are summarized trying to correlate them to chemical, structural and morphological information, when available. Even though, this study cannot be exhaustive because of the lack of a systematic investigation in the literature, this approach can be fundamental for a rational understanding of the optical properties of melanin‐like materials both for a correct interpretation of the behavior of the natural melanin‐based pigments and the development of new better performing artificial devices.

## 2 Different Morphologies and Structures of Melanin

Artificial melanin‐like materials are widely exploited both as models for the understanding of natural melanin and for technological applications.^[17]^ Typically, they are prepared by the oxidation and polymerization of one or more of the molecular precursors listed in Figure 1. Even if the products of this process are often referred as melanin, the kind of nanostructures that can be simultaneously obtained are several and they can evolve during time. For example, a precursor as dopamine (DA) can form, in alkaline environment, polymeric films that are adsorbed efficiently onto almost every kind of surface, but also low and medium molecular weight water soluble species, as well as high molecular weight very monodispersed large NP. Depending on the kind of application and investigation, the various research groups were focused on different products of the process, like on the film instead the NP. For example Messermith and Lee mainly focused on melanin‐coatings,^[14]^ while other research groups, such as Gianneschi's, focused more on melanin‐like NP demonstrating for example their role as powerful agents for photoprotection and radical scavengers[^13^, ^18^] or their use as building block for structural coloration.^[19]^

### 2.1 Melanin NP

Artificial melanin NP are very interesting nanomaterials that efficiently mimic the natural pigment that is present in the human organism in the form of granules, melanosomes.^[13]^ Melanin NP have been prepared from different precursor such as Dopamine, L‐DOPA, 1,8‐dihydroxy naphthalene, DHICA^[3b]^ in the presence of chemical oxidants (in the simplest case atmospheric oxygen in alkaline environment) or by enzymatic oxidation.^[8]^ Although not always clearly mentioned, typically, products other than NP are obtained in the process.[^9a^, ^20^] Regarding the NP, different approaches are exploited to achieve mono‐dispersity and size control of the formed NP. For example, polymerization of DA in ethanol/water mixture in the presence of oxygen and ammonia can lead to size‐controlled polydopamine (PDA) spheres that can be separated simply by centrifugation. In particular, the size of the NP can be controlled by tuning the ratio of ammonia to dopamine.^[21]^ Alternatively, NaOH can be used as a base and in this case the size of the NP can be also controlled by changing the synthesis temperature and the amount of the base.^[22]^ Moreover, specific functionalities, such as TEMPO, can be incorporated in the PDA matrix by copolymerization of DA with specifically functionalized precursors.^[18]^ In the case of L‐DOPA, oxidation by oxygen does not lead to the formation of a relevant fraction of NP and stronger oxidants such as Potassium permanganate (KMnO_4_) are needed for the formation of L‐DOPA NP. Another possibility is the chemo‐enzymatic (tyrosinase and buffer) synthesis of the same type of melanin NP.^[23]^ In addition, KMnO_4_ (or sodium periodate NaIO_4_) has been used for the synthesis of allomelanin NP starting from 1,8‐DHN at ambient temperature.^[24]^ In the case of DHICA, TiO_2_ can be used as a polymerization catalyst for the synthesis of NP.^[25]^ Concerning pheomelanin NP, they can be synthetized either by using as precursors L‐DOPA in combination with tyrosine, either by starting the synthesis from the heterodimer 5‐S‐cysteinyl‐DOPA. In the first case, the synthesis of NP is obtained by mixing L‐DOPA and cysteine in water using KMnO_4_ as an oxidant at room temperature.^[26]^ Starting from the heterodimer, a different synthetic approach is used for the synthesis of NP that are formed in PBS at pH=7.4 and in the presence of different amount of L‐DOPA.^[27]^

**Figure 2:**
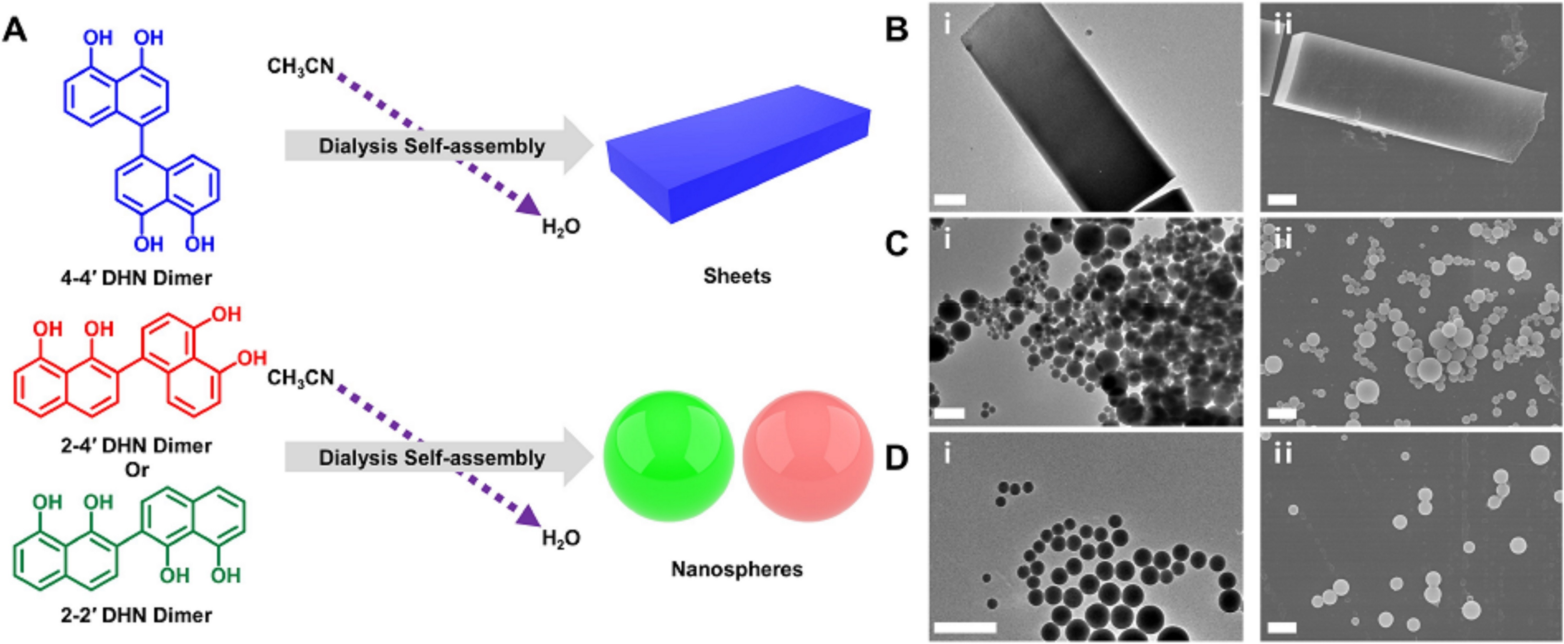
A) Schematic illustration of the dialysis‐mediated self‐assembly of three different 1,8‐dihydroxynaphthalene (DHN) dimers. The supramolecular structure of the three different DHN dimers (4‐4′, 2–4′ and 2–2′) are shown. Their self‐assembled materials are reported as SA. B) (i) TEM image, (ii) scanning electron microscopy (SEM) image of 4–4’‐DHN‐SA. C) (i) TEM image, (ii) SEM image of 2–4’‐DHN‐ SA. D) (i) TEM image, (ii) SEM image of 2–2’‐DHN‐SA using scale bars 600 nm. Reprinted with permission from Ref. [29]. Copyright 2021, Wiley‐VCH.

In order to understand the formation of the NP, the interactions involved in their genesis have been widely discussed.^[28]^ It is interesting to note that Gianneschi's group recently demonstrated that the dimers obtained in a controlled way from an allomelanin precursor do indeed self‐assembly via non‐covalent interaction forming either nanospheres or nanosheets (Figure 2).^[29]^ An important novelty of this investigation is that the formation of purely self‐assembled nanostructure was revealed by proving the reversibility of the process. In fact, the dimers are soluble in acetonitrile (CH_3_CN) and they assemble into large nanostructure, with a morphology which can be controlled to be also anisotropic upon addition of water. In addition, the resulting NP can be disassembled by redissolution in CH_3_CN demonstrating the reversibility of the process and the non‐covalent nature of the interactions. In alternative, the formed structure can be covalently polymerized by ammonia‐induced solid‐state polymerization. Going to enzymatically produced L‐DOPA NP, a new investigation revealed that smaller particles (around 40 nm) aggregate to finally give bigger spherical particles uniform in size and diameter of 200 nm. These aggregates maturate in time increasing their density and the process stops when a final size of 200 nm is reached.^[9b]^ The overall photophysical and photochemical properties of melanin based NP depend both on the nature of the chromophores formed during their growth and on the interchromophoric interactions. The most recent studies for the investigation of these features are based on ultrafast transient absorption spectroscopy, as discussed in section 4.2.

### 2.2 Melanin‐like films

**Figure 3:**
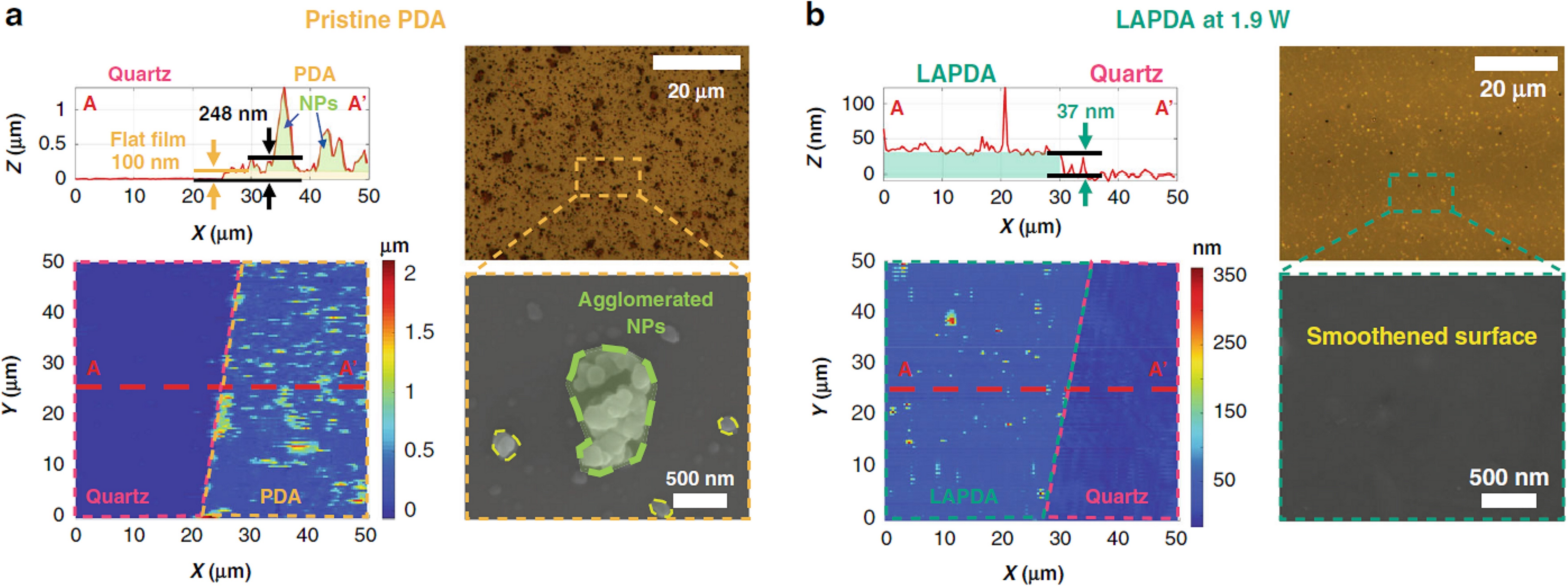
a) Atomic force microscopy (AFM) images with line‐scan profiles, optical microscope images and SEM images of a pristine PDA at 1.9 W and b) AFM images with line‐scan profiles, optical microscope images and SEM images of laser‐annealed PDA at 1.9 W. Reprinted with permission from Ref. [32]. Copyright 2020, Nature portfolio.

Adherent PDA films can be easily formed onto almost any surface by simple dip‐coating of the substrate into a solution of dopamine at alkaline pH.[^14^, ^30^] The deposition kinetics of the PDA film can be affected not only by the nature of the buffer (amine‐containing buffer like Tris or other), but also by the oxidant (oxygen or other).^[31]^ The structure of these films was investigated by Messersmith group, that studied the retraction of a PDA‐coated cantilever from an oxide surface during the coating process. The results revealed the formation of long chain polymers with length up to 200 nm.^[15]^ Although the previous work clearly demonstrated the presence of a polymeric fraction of PDA in the coating solution, it does not completely rule out the possible formation of other species. Indeed, in a more recent work the same authors took into consideration the presence of low molecular weight components in the film, as well as of adsorbed NP hundreds of nanometers in size (Figure 3).^[32]^ The photophysical and photochemical properties of melanin‐based film reflect the presence of monomers, oligomers, polymers and NP as discussed in section 4.

### 2.3 Non‐NP water soluble melanin

Although not always discussed in detail in the literature, depending on the oxidation conditions and the pH, some melanin precursors yield dark water soluble products that share many of the optical properties of melanin and in particular a broad absorption band extended from the UV to the NIR region.^[33]^ A relevant case is the product obtained by the oxidative‐polymerization of L‐DOPA in ammonia solution at pH 8.0^[34]^ that has also been recently investigated in detail by ultrafast transient absorption spectroscopy (UFTA).^[35]^ Importantly, dynamic light Scattering (DLS) analysis of the resulting water solution did not show the presence of any suspended particulate and (transmission electron microscopy) TEM analysis revealed the presence of graphite‐like stacked layers in the dried material. It is interesting to note that in this case the diffraction pattern detected was compatible with an in‐plane periodicity comparable to the spacings in graphene quantum dots and graphite. Moreover, Raman spectroscopy also confirmed the graphene‐like nature of this artificial melanin compound. These observations open a totally new perspective on the possible nature of synthetic melanin but, consequently, also on the natural one. Unfortunately, a more detailed characterization of this water‐soluble melanin directly in solution is still missing. Just as importantly, it should be underlined that also in the case of the synthetic protocols that lead to the formation of melanin‐like NP, only a minor fraction of the product can be isolated in this form. At the same time, after the removal of the NP by centrifugation, most of the reacted precursor remains in solution in a water‐soluble form. We suggest that a more detailed investigation of the photophysical and photochemical properties of these non‐NP melanin would be fundamental to better understand the mechanism of formation of melanin NP.

### 2.4 Low molecular weight products

Although natural melanin has been reported to be a largely insoluble pigment,^[28]^ low molecular weight products of oxidation/condensation of melanin precursors have been detected in various synthetic systems. For example, a significant amount of unpolymerized dopamine has been identified in PDA forming a self‐assembled complex with 5,6‐Dihydroxyindole (DHI).^[9a]^ Indeed, via various characterization techniques the smaller molecular fragments of the synthetic pigment have been recognized, as well as their intermolecular interactions. Fundamentally, proofs of open‐chain dopamine monomeric units and PDA oligomers in a different state of (un)saturation have been reported bibliographically.^[36]^

## 3 Supramolecular Interactions in Melanin

Supramolecular interactions have been reported to play an important role in the formation and stabilization of melanin‐like materials.^[37]^ A strong support to this model is the observation of artificial and natural melanin by high resolution TEM images that showed the presence of aggregates with onion‐like nanostructures resulting from stacked planes arranged in concentric rings.^[38]^ The spatial period obtained by Fourier analysis was 3.7–3.9 Å depending on the position in the onion‐like structure. Chen et al. explained this result by simulating the self‐assembly of tetramers of 5,6‐dihydroxyindole showing that these molecules tend to give non‐covalent π‐π stacking secondary structures, with an interlayer distance of about 3.3 Å. The self‐assembly of molecular precursors has been also demonstrated in solution by techniques as liquid chromatography, mass spectrometry and spectrophotometric analysis,^[39]^ and has been predicted by simulation.^[40]^ Typical patterns arising from supramolecular interactions involved in melanin‐related materials are schematized in Figure 1 i‐k. Regarding the formation of NP, the hierarchical structure of melanin has been demonstrated following the growth of artificial melanin NP. According to Strube and coworkers, the formation of melanin NP is the result of a four‐level process that starts from i) the formation of oligomers and then follows ii) the aggregation in proto‐particles of 1 nm size; iii) the assembly of the proto‐particles into type‐A particles (size 10 nm) and iv) the final formation of type‐B particles of 100 nm.[^9b^, ^41^]

## 4 Photophysics and Photochemistry

### 4.1 Light extinction properties

As it is well known, eumelanin in solution, but also in the condensed phase, possesses a characteristic monotonic broad band UV‐Vis absorption spectrum which extends also to the NIR region.^[10]^ However, quantifying the actual ability of melanin to absorb light at the different wavelengths is really important. A first critical point is that the molar absorption coefficient, ϵ, typically used for molecules, can be applied to melanin only in solution and it requires the ability to determine the molar concentration, that is a difficult accessible information. Nevertheless, some attempts for the understanding of the light‐related parameters of melanin have been reported bibliographically. To begin with, Piletic et al. reported at 750 nm an ϵ=1500 and 3200 M^−1^cm^−1^ for Sepia eumelanin and synthetic pheomelanin respectively.^[42]^ In the case of films, Bothma et al. reported for synthetic eumelanin an absorption coefficients, α, of 9×10^6^–1.3×10^6^ m^−1^ from 400 to 800 nm.[^7d^, ^43^] In the case of melanin NP, also light attenuation needs to be considered due to scattering. These NP have a relatively high refractive index taking into consideration that conventionally an arbitrary value of 2.0 was used for optical simulations of the refractive index.^[44]^ Nevertheless, the exact value of refractive index is still debated and it almost certainly varies between different melanin chemistries. Kurtz et al., for example reported for sepia‐ink melanin a refractive index of 1.66 at the wavelength of 633 nm.^[45]^ In general, the extinction spectrum (that considers both light absorption and scattering) of melanin‐like materials is relatively independent on the particle size.^[22]^ In fact, although Rayleigh and Mie scattering have to be considered, it was found that scattering contributes less than 6 % to the optical attenuation^[46]^ and electronic transitions mostly contribute to light extinction.^[35]^ Nevertheless, the actual nature of the electronic transitions involved in melanin is still largely unexplored. Regarding this, the possible presence of charge‐transfer transition was recently, in part, ruled out since reductive treatment was reported not to affect the absorption spectrum of the bigger sub‐units in the case of PDA NP.^[22]^

### 4.2 Ultrafast transient absorption

**Figure 4:**
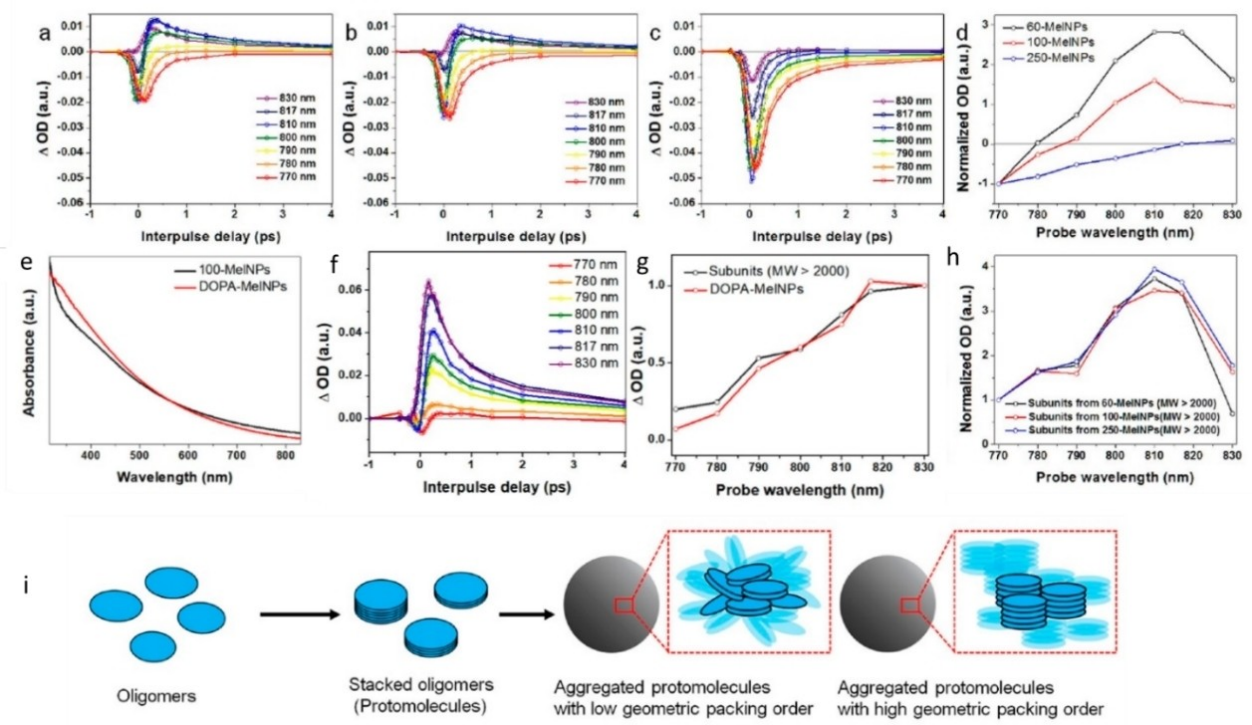
a) Transient absorption signal of PDA NP 60 nm b) transient absorption signal of PDA NP 100 nm c) transient absorption signal of PDA NP 250 nm. All a, b and c are obtained with excitation pump at 720 nm and probe wavelength ranging from 770 to 830 nm. d) Time‐delay signal of the three different size PDA NP (60 nm, 100 nm, 250 nm) at 1 ps as a function of the probe wavelength. The signals are normalized at 770 nm. e) UV‐Vis Absorbance spectra of 100 nm PDA NP and of L‐DOPA NP. f) Transient absorption signals of L‐DOPA NP using 720 nm pump and 770–830 nm probe. g) Time‐delayed transient signal of L‐DOPA NP and their high molecular weight subunits at 1 ps as a function of probe wavelength. The signal is normalized at 830 nm. h) Time‐delay signal of the high molecular weight subunit fractions deriving from the oxidation of the three different PDA NP (60 nm,100 nm,250 nm) at 1 ps as a function of probe wavelength. The signal is normalized at 770 nm. i) Proposed relationship between structure and properties of eumelanin based on the hierarchical assembly structure. Reprinted with permission from Ref. [22].Copyright 2018, ACS Publications.

Photophysical processes following light absorption by melanin‐like materials occur in the scale of femto to nano‐seconds.^[47]^ Ultrafast transient absorption spectroscopy (UFTA) is hence fundamental for the understanding^[48]^ of the connection between composition, structure and the physicochemical properties of melanin and it was proposed for the identification of the possible differences between the various artificial pigments and the natural one. Initially, research was mainly focused on the different photoreactivity of pheomelanin and eumelanin, already reported in 1980,^[49]^ and was further investigated using UFTA some years after in both the UV and the Vis region comparing naturally extracted sepiamelanin to enzymatically produced synthetic pheomelanin.^[50]^ Based on UFTA, it was hypothesized that artificial pheomelanin can aggregate in structures of different size which are responsible for the multi‐exponential dynamics, while the transient species was recognized as the S_1_ excited state.^[50a]^ In further studies, low molecular weight fractions of pheomelanin showed similar photophysics to the aggregated pheomelanin, but different absorption spectrum from the bulk pigment.^[50c]^ When human‐extracted pheomelanosomes and eumelanosomes were tested, it was found that pheomelanin is more prone to cause cellular damage through UV‐induced ionization.^[50d]^ Even if the harsh chemical conditions may alter the pigment after the extraction, studies on DNA have also demonstrated that indeed photoreactions of melanocytes are involved in the formation of long lived excited states.^[51]^ In some cases, the photochemistry of eumelanin was interpreted through the study of catechol and their derivatives that are considered to be the building blocks of the pigment.^[52]^ More in detail, the intermolecular hydrogen bonding between the units of eumelanin was explained using o‐quinone heterodimer as a model.^[52a]^ By selectively exciting only the o‐quinone of the heterodimer at 365 nm, the singlet excited state formed could cause photoinduced hydrogen transfer to the catechol leading to semiquinone radical pairs. In a picosecond (ps) time scale, the same could also occur after inter‐system‐crossing from the excited triplet. In both cases, a long‐lived radical pair was formed which did not recombine after 2.5 ns. In another work, the effect of aggregation was investigated using 4‐t‐butylcatechol as a model.^[52b]^ This molecule does not spontaneously oxidize in solution, but it tends to aggregate in organic solvent at high concentration forming hydrogen bonds. According to this study, the aggregation significantly affected the photophysics of the precursor as the photoinduced S_1_ tended to decay more slowly in the aggregated structure than in the monomer, thus exhibited a longer lifetime. Going beyond model systems, Kohler's group recently revealed the great chemical heterogeneity of non‐particulate L‐DOPA melanin, which dynamics were studied in a very broad spectral window from the UV to the Vis.^[35]^ The results showed a constant spectral shape upon pumping at 265 nm, which was not alike the transient absorption spectrum of DHI. Going towards the Vis, a significant in magnitude, persistent in time, spectral hole was formed centered on the pump wavelength. This hole formation was attributed to the selective excitation of melanin chromophores which are nearby in energy. CT (charge transfer) exciton formation in less than 200 fs was found to describes better the dynamics of the pigment. For these characteristics, this artificial melanin was compared to disordered carbonaceous nanomaterials that form immobile charge transfer excitons in less than 200 fs. The same group investigated further the same type of artificial melanin by transient vibrational spectroscopy in the double bond stretching region.^[53]^ Using UV‐Vis pump (265‐600 nm) for selective excitation of the different melanin chromophores, and mid‐IR probe pulse for the analysis of the transient changes in vibrational modes, these authors found that even if different chromophores possess different absorption, the number and type of IR‐active functional groups were the same demonstrating that: i) melanin chromophores that absorb in different wavelength cannot just differ in the oxidation state; ii) same‐size aggregates cannot differ just in the functional groups because this would result in different IR signatures. The results were explained assuming electronic coupling of closely spaced chromophores, which can lead to wavelength‐independent excited state relaxation. Another interesting study aimed to correlate melanin NP composition and size to optical properties. Warren and coworkers compared the optical properties of dopamine (DA)‐bases melanin NP of three different sizes (∼60 nm, ∼100 nm and ∼250 nm diameter) obtained by oxidation of DA with atmospheric oxygen in the presence of NaOH.^[22]^ These NP presented a size‐dependent steady state absorption spectrum and, as shown in Figure 4a‐4c, different excited‐state dynamics as analyzed by UFTA microscopy. Moreover, as shown in Figure 4f, the transient spectral features of L‐DOPA NP (d∼100 nm), obtained by oxidation with KMnO_4_, were different from those of ∼100 nm DA NP suggesting that the nature of the precursor and of the oxidant affect the photophysical behavior of the NP. In a next step, the effect of the disassembly upon hydrolysis (in the absence of oxygen) of the four kinds of NP in NaOH was investigated. The alkaline treatment led in all the cases to the formation of low molecular weight components (MW<2000) and of higher molecular weight products that showed in AFM sizes of 0.2‐2 nm. Interestingly, the transient spectrum of the fragments was similar to the one of the NP only for L‐DOPA and not for DA.

### 4.3 Photoluminescence and photothermal properties

Melanin‐like materials are considered not only to be non‐fluorescent, but also to be fluorescence quenchers when used in combination with other dyes (FRET/PET).^[54]^ This effect can be attributed to the fact that the planar aromatic rings self‐assemble in the non‐covalent structure via intra or/and intermolecular stacking producing aggregation‐caused quenching (ACQ). Hence, excited melanin mostly deactivates via non‐radiative paths producing local photothermal heating, a phenomenon that has been widely explored in nanomedicine and photothermal catalysis and has been discussed in recent review papers.^[55]^ Since a lower degree of π‐π interactions is expected to lead to increased fluorescence, several strategies have been proposed to rise the emission of melanin‐like materials by modifying and/or reducing π‐π stacking interaction. In particular, Yang et al. summarized chemical oxidation, degradation, conjugation and carbonization as the most common methods to obtain fluorescent melanin‐like materials.^[7b]^ It is interesting to note that the nature of the precursor alters the fluorescence of the produced melanin‐like materials. As an example, a relevant difference in the presence of proton transfer pathways was highlighted in DICHA and DHI precursors.^[3b]^ Moreover, Corani and co‐workers studied the behavior of 5,6‐dihydroxyindole (DHI) and 5,6‐dihydroxyindole‐2‐carboxylic acid (DICHA) oligomers by ultrafast fluorescence spectroscopy.^[56]^ They observed an excited state deactivation 3 order of magnitude faster in DICHA than in DHI due to the intra and interunit proton transfer, occurring exclusively in DICHA structure. In another case, Lin et al. synthesized fluorescent PDA dots (PDs) by a hydroxyl radical‐mediated degradation of PDA NP obtaining blue and green emitting PDs, measuring the fluorescence lifetime.^[57]^ According to the authors, the shorter lifetime of the green emitting PDs (9.4 ns) with respect to the blue ones (10.8 ns) denoted a higher oligomerization and self‐assembly in the former than in the latter. Fluorescent PDA was also obtained by the polymerization of DA in the presence of a co‐reactant, as glutathione (GSH), in order to reduce aggregation‐caused quenching effect (ACQ) and obtain chemosensors for Cu^2+^ and Fe^3+^.^[58]^ This approach was reported to be more advantageous than the oxidation with H_2_O_2_ since the initially achieved fluorescence is lost after NP purification. Other Fe^3+^ sensors, designed by Yin and co‐workers, were based on blue emitting PDA NP obtained by modulating their surface redox properties.^[59]^ Liu et al. obtained fluorescent PDA for Zn^2+^ detection using a nanozyme assisted oxidation method in two steps: i) polymerization of dopamine under basic conditions ii) dopamine peroxidation catalyzed by the nanozyme leading to the final fluorescent product.^[60]^ In another case, a PDA‐based fluorescent sensors for reduced glutathione was proposed using MnO_2_ as oxidizing agent to synthesize PDA fluorescent NP. By exploiting the capability of GSH to reduce MnO_2_ to Mn^2+^ it was thus possible to selectively inhibit the formation of fluorescent PDA.^[61]^ Lastly, Quignard and co‐workers proposed the synthesis of fluorescent PDA NP by exploiting photo‐oxidation of DA using oil microdroplets as templates.^[62]^ In general, it should be stressed that although several approaches have been developed to convert melanin NP into fluorescent species: i) it is not clearly demonstrated that the generated fluorescence arises from the modified melanin NP and not from molecular side‐products; ii) the actual mechanism at the basis of the fluorescence is widely unknown.

### 4.4 Photoreactivity: photochemical or photothermal?

**Figure 5:**
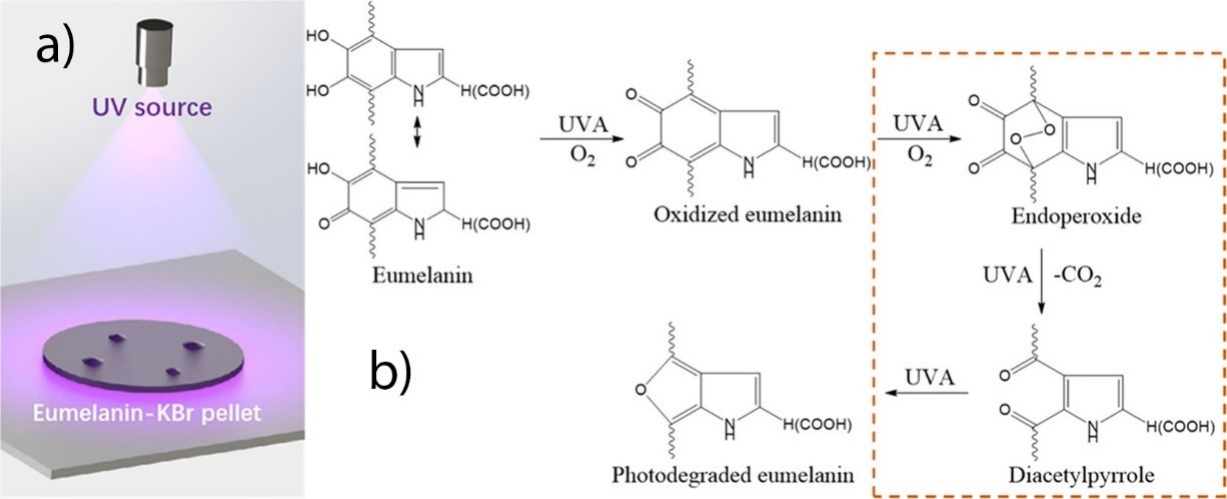
a) Schematic illustration of irradiation of the compressed eumelanin‐KBr pellet using UVA source with wavelength range from 320 to 450 nm with an intensity at the sample surface of 2.43±0.02 W/cm^2^. b) proposed chemical for the UV‐induced oxidation of eumelanin pellets leading to the release of CO_2_. Adapted with permission from Ref. [7c]. Copyright 2019, ACS Publications

Within the light‐induced processes in melanin‐like materials are included photochemical reactions (involving excited states) or photothermal processes (chemical processes involving the ground state and activated by photothermal heating).^[63]^ Nevertheless, this difference is rarely considered in the scientific literature. In a recent paper Li et al. investigated the mechanism of photochemical degradation of three different kinds of melanin NP under UV irradiation using solid‐state NMR and FTIR spectroscopy.^[7c]^ The irradiation was carried out on dried samples of two synthetic melanin NP samples, made from dopamine (d=100±11 nm) and L‐DOPA (d=183±13 nm), and one natural Sepia melanin (d=100‐150 nm^[64]^) sample. This study demonstrated that while under UVA irradiation no change in the pyrrole ring of the indole unit of PDA was observed, on the contrary a fraction of the six‐membered benzyl ring was broken leading to furo[3,4‐b]pyrrole. In particular, it has been suggested that the oxidation of the tautomers DHI or DHICA to quinone structures led to the formation of furans able to release CO_2_ (Figure 5). Interestingly, this investigation showed that the photochemical pathway is the same in all the three NP samples, despite the different composition and natural or artificial origin. Although, no information about the effect of the irradiation on the NP integrity or morphology was given.

As far as melanin‐like films is concerned, Messersmith and coworkers recently reported how PDA coating can be strengthen by photo‐treatment by scanning the threated surface with a focused blue laser diode.^[32]^ Importantly, light irradiation did not compromise the functionality of the surface and led to a decrease of the porosity that, accordingly to the author, was strongly related to the presence of agglomerated NP on the surface. The previous means that also in the case of coating, the produced film contains: PDA polymers, dopamine monomer and oligomers with low molecular weight (identified by MALDI‐TOF) and PDA NP (d∼200 nm). The effect of the irradiation on the different components was reported to be very different. In fact, while the continuous polymeric film underwent graphitization, as confirmed by Raman spectroscopy, the protruding NP were ablated by the laser beam producing a more regular and flatter surface (Figure 3). These results clearly demonstrated the very different photo‐reactivity of PDA films and PDA NP proving that indeed the morphology plays in this framework a fundamental role. Unfortunately, according to the previous results it is not possible to identify completely the occurring light‐activated process and even more importantly, what is the contribution of the photochemical and photothermal processes involved.

## 5 Conclusions and Perspectives

Melanin‐like materials are continuously finding new light‐based applications in fields of high economic and social impact including medicine, energy conversion and photocatalysis. Nevertheless, the understanding of the photophysics and photochemistry of these materials is still incomplete. In this minireview we underlined that a more generalized and systematic approach to correlate the photophysical and photochemical properties of melanin‐like materials to their chemical structure and to their morphology is needed. In particular, we discussed in detail how melanin‐like materials typically represent a combination of families of different species (varying from monomer or oligomer to polymer and NP) with analogous, but, in some cases, also very different properties. Hence, the overall photophysical and photochemical features depend, in part, on these of the chromophores contained in this variety of species. However, as typical for supramolecular systems, these properties are, to a large extent, altered by inter‐chromophoric interactions.^[65]^ This work highlights the most recent and relevant scientific papers (further summary is given in Table 1), where this issue has been analyzed in detail. Between the different techniques used for the optical characterization of melanin, UFTA is surely one of the most promising since it allows the investigation of the various photo‐induced fast processes occurring in melanin‐like materials. Nevertheless, it should be stressed that, even if important information is available in the literature, the following points deserve further investigation: i) what is the nature of the excited states produced upon ultrafast excitation; ii) if and how efficiently interconversion between different excited states occurs and iii) how the nature of the excited states and their dynamics are affected by the chemical composition and morphology. We believe that a detailed investigation of these features presents a real challenge for future research in the file of melanin‐like materials. Similarly, understanding the actual origin of the fluorescence induced in these materials is fundamental. In particular, in the case of melanin NP it has not been definitively demonstrated whether the fluorescence comes from the NP themselves or from molecular residuals produced during the treatments applied in order to produce emission. As far as the photo‐reactivity is concerned, examples of evidence of chemical modification of melanin via light irradiation have been also reported. This is a very important result taking into consideration the role of melanin in photoprotection that requires, as a further improvement, an extensive characterization of the photoproduct, as well as a quantification of the quantum yield. More in general, we believe that the strategy followed in this paper is aimed at differentiating the various kinds of materials classified as melanin and at investigating the specific photophysical and photochemical properties correlated to each type, that are essential for the understanding of the basic properties of these materials. Additionally, this strategy is going to aid in a better and controlled design of new materials for technological application.

**Table 1:** Correlating the morphology and structure of melanin to the main references where their photophysical and photochemical properties are discussed.

Structure/morphology	Main Ref.
Monomers/Oligomers	[11b], [22], [35], [40b], [52a], [52b]
Polymers	[15], [32], [35], [53]
NP	[7c], [13], [18], [21b], [22], [24], [32], [50g]
Films	[19], [32]

## Conflict of interest

The authors declare no conflict of interest.

## Biographical Information

*Alexandra Mavridi‐Printezi graduated in Chemistry (Advanced Cosmetic Science) and she is now a PhD student in the group of Prof. Marco Montalti. Her main research activity is the development of new melanin based photoactive supramolecular and/or nanostructured platforms for applications to energy conversion, environmental remediation and ROS photogeneration. She is also involved in the development of new materials for nano‐cosmetics*.

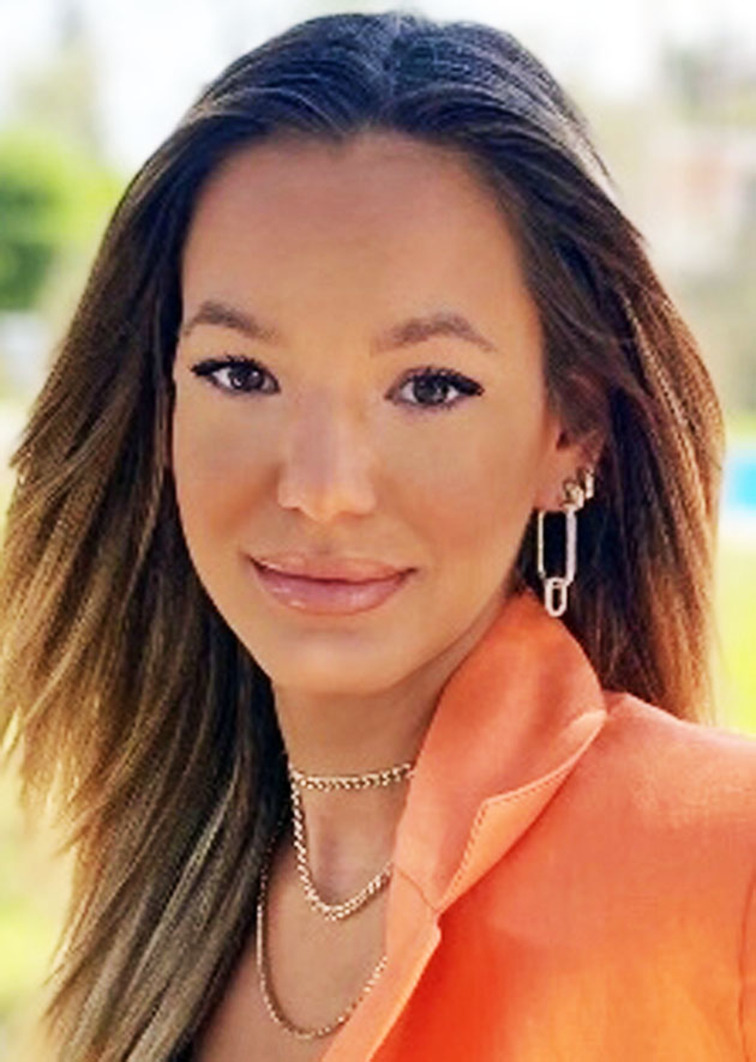

## Biographical Information

*Moreno Guernelli graduated in Photochemistry and Molecular Materials and he is now a PhD student in the group of Prof. Marco Montalti. His research is mostly devoted to photoactive coatings for decontamination of real surfaces (e. g. cement, steel, glass and wood) from bacteria and viruses. He studied new photo‐sensitizers to extend the photocatalytic activity of metal‐oxide nanoparticles to the visible and near‐infrared*.

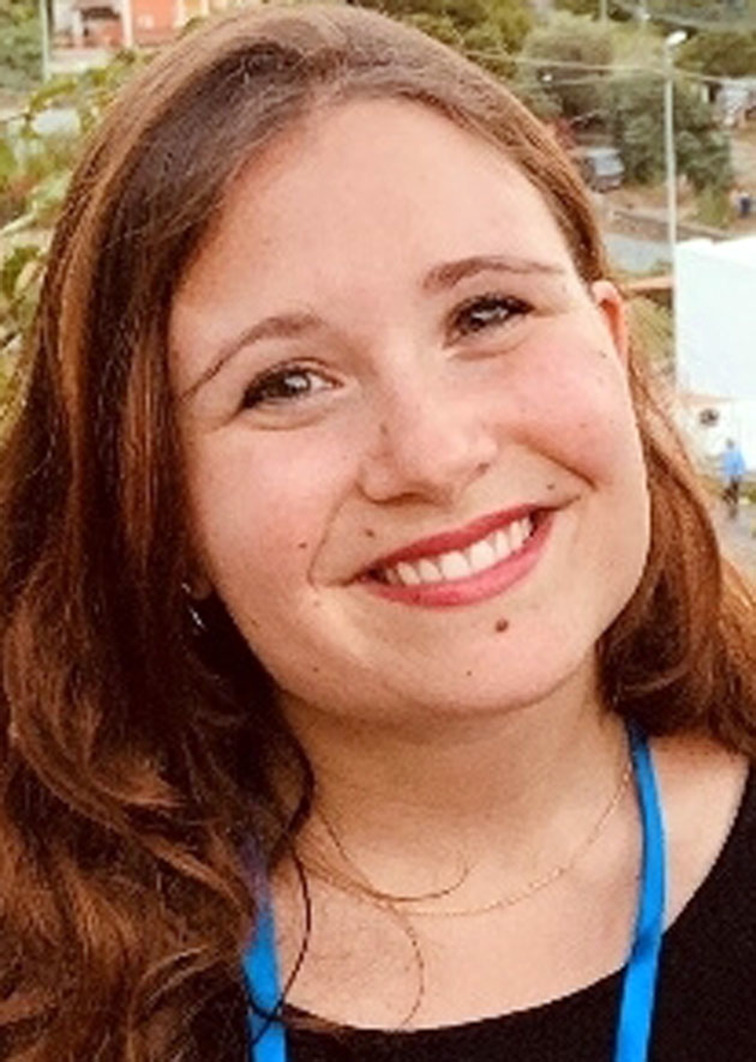

## Biographical Information

*Arianna Menichetti graduated in Photochemistry and Molecular Materials and she is now a PhD student in the group of Prof. Marco Montalti. Her main research activity is the development of photoactive precursors for the nanofabrication of inorganic materials by 3D laser printing. Applications of these materials include tissue regeneration, energy conversion, photocatalysis and photo‐electronics*.

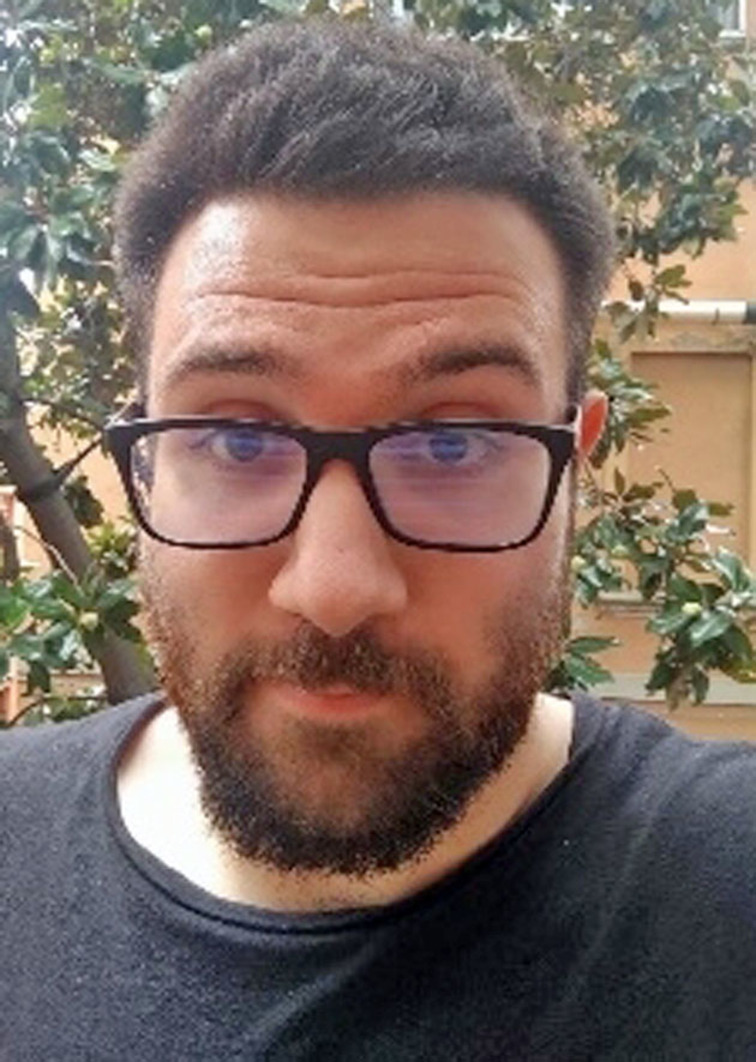

## Biographical Information

*Marco Montalti is Professor of Chemistry at the University of Bologna. The main research topics of his group are the design, production, and characterization of Vis‐NIR photoactive and/or stimuli responsive supramolecular and nanostructured architectures and nanocomposites. Applications of these materials include photocatalysis for energy conversion and environmental remediation, bioimaging and theragnostic*.

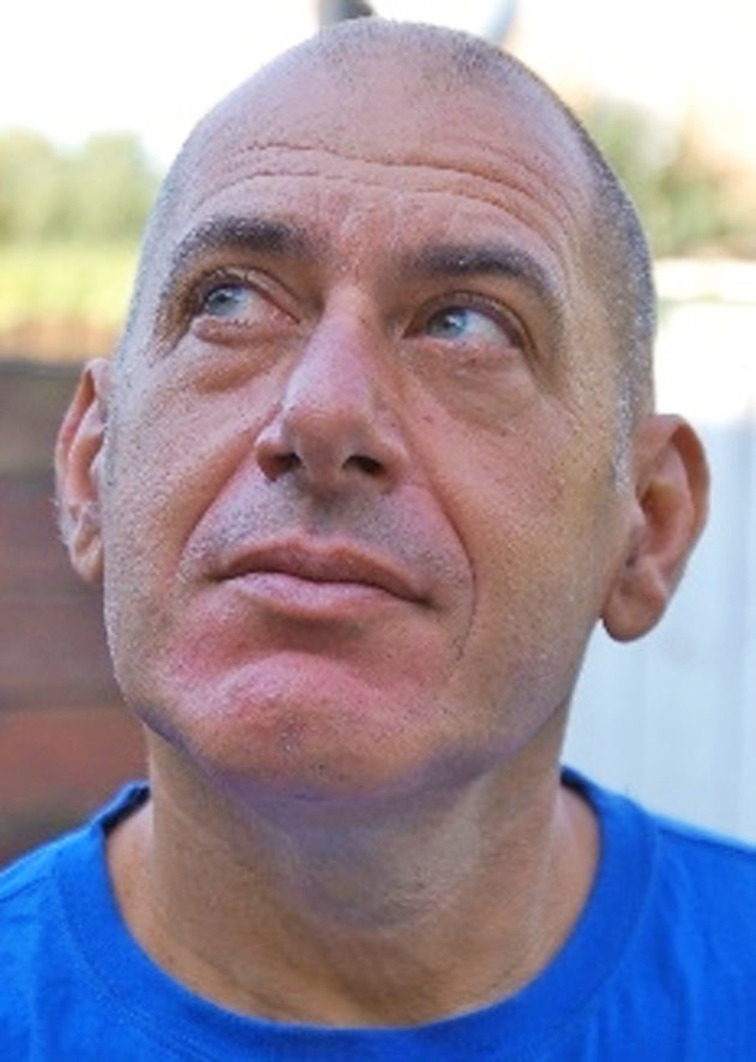

## References

[1a] I. Galván , F. Solano , Int. J. Mol. Sci. 2016, 17, 520; 27070583 10.3390/ijms17040520 PMC4848976

[1b] M. d′Ischia , K. Wakamatsu , F. Cicoira , E. Di Mauro , J. C. Garcia-Borron , S. Commo , I. Galván , G. Ghanem , K. Kenzo , P. Meredith , A. Pezzella , C. Santato , T. Sarna , J. D. Simon , L. Zecca , F. A. Zucca , A. Napolitano , S. Ito , Pigm. Cell Melanoma Res. 2015, 28, 520–544. 10.1111/pcmr.12393 26176788

[2] J. D‘Orazio , S. Jarrett , A. Amaro-Ortiz , T. Scott , Int. J. Mol. Sci. 2013, 14, 12222–12248. 23749111 10.3390/ijms140612222 PMC3709783

[3a] M. d′Ischia , A. Napolitano , A. Pezzella , P. Meredith , M. Buehler , Angew. Chem. Int. Ed. 2020, 59, 11196–11205; 10.1002/anie.201914276 31867862

[3b] W. Cao , X. Zhou , N. C. McCallum , Z. Hu , Q. Z. Ni , U. Kapoor , C. M. Heil , K. S. Cay , T. Zand , A. J. Mantanona , A. Jayaraman , A. Dhinojwala , D. D. Deheyn , M. D. Shawkey , M. D. Burkart , J. D. Rinehart , N. C. Gianneschi , J. Am. Chem. Soc. 2021, 143, 2622–2637. 33560127 10.1021/jacs.0c12322

[4] I.-E. Pralea , R.-C. Moldovan , A.-M. Petrache , M. Ilieş , S.-C. Hegheş , I. Ielciu , R. Nicoară , M. Moldovan , M. Ene , M. Radu , A. Uifălean , C.-A. Iuga , Int. J. Mol. Sci. 2019, 20, 3943. 10.3390/ijms20163943 PMC6719904 31412656

[5a] M. Caldas , A. C. Santos , F. Veiga , R. Rebelo , R. L. Reis , V. M. Correlo , Acta Biomater. 2020, 105, 26–43; 32014585 10.1016/j.actbio.2020.01.044

[5b] Y. Liu , K. Ai , L. Lu , Chem. Rev. 2014, 114, 5057–5115; 24517847 10.1021/cr400407a

[5c] W. Xie , E. Pakdel , Y. Liang , Y. J. Kim , D. Liu , L. Sun , X. Wang , Biomacromolecules 2019, 20, 4312–4331. 31696698 10.1021/acs.biomac.9b01413

[6] N. C. McCallum , F. A. Son , T. D. Clemons , S. J. Weigand , K. Gnanasekaran , C. Battistella , B. E. Barnes , H. Abeyratne-Perera , Z. E. Siwicka , C. J. Forman , X. Zhou , M. H. Moore , D. A. Savin , S. I. Stupp , Z. Wang , G. J. Vora , B. J. Johnson , O. K. Farha , N. C. Gianneschi , J. Am. Chem. Soc. 2021, 143, 4005–4016. 33673734 10.1021/jacs.1c00748

[7a] A. Mavridi-Printezi , M. Guernelli , A. Menichetti , M. Montalti , Nanomaterials 2020, 10; 10.3390/nano10112276 PMC7698489 33212974

[7b] P. Yang , S. Zhang , X. Chen , X. Liu , Z. Wang , Y. Li , Mater. Horiz. 2020, 7, 746–761;

[7c] W. Li , Z. Wang , M. Xiao , T. Miyoshi , X. Yang , Z. Hu , C. Liu , S. S. C. Chuang , M. D. Shawkey , N. C. Gianneschi , A. Dhinojwala , Biomacromolecules 2019, 20, 4593–4601; 31696706 10.1021/acs.biomac.9b01433

[7d] M. Xiao , M. D. Shawkey , A. Dhinojwala , Adv. Opt. Mater. 2020, 8, 2000932.

[8] J. Liebscher , Eur. J. Org. Chem. 2019, 2019, 4976–4994.

[9a] S. Hong , Y. S. Na , S. Choi , I. T. Song , W. Y. Kim , H. Lee , Adv. Funct. Mater. 2012, 22, 4711–4717;

[9b] A. Büngeler , B. Hämisch , K. Huber , W. Bremser , O. I. Strube , Langmuir 2017, 33, 6895–6901. 28639791 10.1021/acs.langmuir.7b01634

[10] P. Meredith , T. Sarna , Pigm. Cell Res. 2006, 19, 572–594. 10.1111/j.1600-0749.2006.00345.x 17083485

[11a] J. I. N. Cheng , S. C. Moss , M. Eisner , Pigm. Cell Res. 1994, 7, 263–273; 10.1111/j.1600-0749.1994.tb00061.x 7855075

[11b] G. G. B. Alves , F. C. Lavarda , C. F. O. Graeff , A. Batagin-Neto , J. Mol. Graphics Modell. 2020, 98, 107609. 10.1016/j.jmgm.2020.107609 32305687

[12] Q. Lyu , N. Hsueh , C. L. L. Chai , Polym. Chem. 2019, 10, 5771–5777.

[13] Y. Huang , Y. Li , Z. Hu , X. Yue , M. T. Proetto , Y. Jones , N. C. Gianneschi , ACS Cent. Sci. 2017, 3, 564–569. 28691067 10.1021/acscentsci.6b00230 PMC5492417

[14] H. Lee , S. M. Dellatore , W. M. Miller , P. B. Messersmith , Science 2007, 318, 426. 17947576 10.1126/science.1147241 PMC2601629

[15] P. Delparastan , K. G. Malollari , H. Lee , P. B. Messersmith , Angew. Chem. Int. Ed. 2019, 58, 1077–1082; 10.1002/anie.201811763 PMC6424361 30485624

[16a] M. Matta , A. Pezzella , A. Troisi , J. Phys. Chem. Lett. 2020, 11, 1045–1051; 31967830 10.1021/acs.jpclett.9b03696

[16b] L. Panzella , G. Gentile , G. D′Errico , N. F. Della Vecchia , M. E. Errico , A. Napolitano , C. Carfagna , M. d′Ischia , Angew. Chem. Int. Ed. 2013, 52, 12684–12687; 10.1002/anie.201305747 24123614

[16c] G. Prampolini , I. Cacelli , A. Ferretti , RSC Adv. 2015, 5, 38513–38526.

[17a] H. Liu , Y. Yang , Y. Liu , J. Pan , J. Wang , F. Man , W. Zhang , G. Liu , Adv. Sci. 2020, 7, 1903129; 10.1002/advs.201903129 PMC7141020 32274309

[17b] R. M. Haywood , M. Lee , C. Linge , J. Photochem. Photobiol. B 2006, 82, 224–235. 16446096 10.1016/j.jphotobiol.2005.12.007

[18] W. Cao , A. J. Mantanona , H. Mao , N. C. McCallum , Y. Jiao , C. Battistella , V. Caponetti , N. Zang , M. P. Thompson , M. Montalti , J. F. Stoddart , M. R. Wasielewski , J. D. Rinehart , N. C. Gianneschi , Chem. Mater. 2020, 32, 5759–5767.

[19] M. Xiao , Y. Li , M. C. Allen , D. D. Deheyn , X. Yue , J. Zhao , N. C. Gianneschi , M. D. Shawkey , A. Dhinojwala , ACS Nano 2015, 9, 5454–5460. 25938924 10.1021/acsnano.5b01298

[20] K.-Y. Ju , Y. Lee , S. Lee , S. B. Park , J.-K. Lee , Biomacromolecules 2011, 12, 625–632. 21319809 10.1021/bm101281b

[21a] K. Ai , Y. Liu , C. Ruan , L. Lu , G. Lu , Adv. Mater. 2013, 25, 998–1003; 23239109 10.1002/adma.201203923

[21b] X. Wang , Z. Chen , P. Yang , J. Hu , Z. Wang , Y. Li , Polym. Chem. 2019, 10, 4194–4200.

[22] K.-Y. Ju , M. C. Fischer , W. S. Warren , ACS Nano 2018, 12, 12050–12061. 30500158 10.1021/acsnano.8b04905

[23] M. Vij , R. Grover , V. Gotherwal , N. A. Wani , P. Joshi , H. Gautam , K. Sharma , S. Chandna , R. S. Gokhale , R. Rai , M. Ganguli , V. T. Natarajan , Biomacromolecules 2016, 17, 2912–2919. 27477067 10.1021/acs.biomac.6b00740

[24] X. Zhou , N. C. McCallum , Z. Hu , W. Cao , K. Gnanasekaran , Y. Feng , J. F. Stoddart , Z. Wang , N. C. Gianneschi , ACS Nano 2019, 13, 10980–10990. 31524373 10.1021/acsnano.9b02160

[25] G. Vitiello , A. Pezzella , A. Zanfardino , B. Silvestri , P. Giudicianni , A. Costantini , M. Varcamonti , F. Branda , G. Luciani , Mater. Sci. Eng. C 2017, 75, 454–462. 10.1016/j.msec.2016.12.135 28415485

[26] J. Pyo , K.-Y. Ju , J.-K. Lee , J. Photochem. Photobiol. B 2016, 160, 330–335. 27173400 10.1016/j.jphotobiol.2016.04.022

[27] G. Greco , L. Panzella , G. Gentile , M. E. Errico , C. Carfagna , A. Napolitano , M. d′Ischia , Chem. Commun. 2011, 47, 10308–10310. 10.1039/c1cc13731j 21858305

[28] M. d′Ischia , A. Napolitano , A. Pezzella , P. Meredith , T. Sarna , Angew. Chem. Int. Ed. 2009, 48, 3914–3921; 10.1002/anie.200803786 PMC2799031 19294706

[29] X. Zhou , X. Gong , W. Cao , C. J. Forman , J. Oktawiec , L. D′Alba , H. Sun , M. P. Thompson , Z. Hu , U. Kapoor , N. C. McCallum , C. D. Malliakas , O. K. Farha , A. Jayaraman , M. D. Shawkey , N. C. Gianneschi , Angew. Chem. Int. Ed. 2021, 60,17464. 10.1002/anie.202103447 33913253

[30a] L. C. Almeida , T. Frade , R. D. Correia , Y. Niu , G. Jin , J. P. Correia , A. S. Viana , Sci. Rep. 2021, 11, 2237; 33500469 10.1038/s41598-021-81816-1 PMC7838280

[30b] S. Mei , X. Xu , R. D. Priestley , Y. Lu , Chem. Sci. 2020, 11, 12269–12281. 34094435 10.1039/d0sc04486e PMC8162453

[31] F. Bernsmann , V. Ball , F. Addiego , A. Ponche , M. Michel , J. J. d. A. Gracio , V. Toniazzo , D. Ruch , Langmuir 2011, 27, 2819–2825. 21332218 10.1021/la104981s

[32] K. Lee , M. Park , K. G. Malollari , J. Shin , S. M. Winkler , Y. Zheng , J. H. Park , C. P. Grigoropoulos , P. B. Messersmith , Nat. Commun. 2020, 11, 4848. 32973166 10.1038/s41467-020-18654-8 PMC7515926

[33] M. d'Ischia , A. Napolitano , V. Ball , C.-T. Chen , M. J. Buehler , Acc. Chem. Res. 2014, 47, 3541–3550. 25340503 10.1021/ar500273y

[34] A. B. Mostert , K. J. P. Davy , J. L. Ruggles , B. J. Powell , I. R. Gentle , P. Meredith , Langmuir 2010, 26, 412–416. 20038178 10.1021/la901290f

[35] F. R. Kohl , C. Grieco , B. Kohler , Chem. Sci. 2020, 11, 1248–1259. 10.1039/c9sc04527a PMC8148383 34123249

[36] J. Liebscher , R. Mrówczyński , H. A. Scheidt , C. Filip , N. D. Hădade , R. Turcu , A. Bende , S. Beck , Langmuir 2013, 29, 10539–10548. 23875692 10.1021/la4020288

[37] A. A. R. Watt , J. P. Bothma , P. Meredith , Soft Matter. 2009, 5, 3754–3760.

[38] C.-T. Chen , V. Ball , J. J. de Almeida Gracio , M. K. Singh , V. Toniazzo , D. Ruch , M. J. Buehler , ACS Nano 2013, 7, 1524–1532. 23320483 10.1021/nn305305d

[39a] W. Chan , Rapid Commun. Mass Spectrom. 2019, 33, 429–436; 30556629 10.1002/rcm.8373

[39b] Y. Li , J. Liu , Y. Wang , H. W. Chan , L. Wang , W. Chan , Anal. Chem. 2015, 87, 7958–7963. 26153916 10.1021/acs.analchem.5b01837

[40a] S. Supakar , A. Banerjee , T. Jha , Comput. Theor. Chem. 2019, 1151, 43–49;

[40b] P. Ghosh , D. Ghosh , J. Phys. Chem. B 2021, 125, 547–556. 33410319 10.1021/acs.jpcb.0c10555

[41] A. Büngeler , B. Hämisch , O. I. Strube , Int. J. Mol. Sci. 2017, 18,1901. 10.3390/ijms18091901 PMC5618550 28878140

[42] I. R. Piletic , T. E. Matthews , W. S. Warren , J. Chem. Phys. 2009, 131, 181106–181106. 19916591 10.1063/1.3265861 PMC4108625

[43] J. P. Bothma , J. de Boor , U. Divakar , P. E. Schwenn , P. Meredith , Adv. Mater. 2008, 20, 3539–3542.

[44] M. Xiao , A. Dhinojwala , M. Shawkey , Opt. Express. 2014, 22, 14625–14636. 24977558 10.1364/OE.22.014625

[45] A. R. Haake , G. A. Scott , J. Invest. Dermatol. 1991, 96, 71–76. 1898964 10.1111/1523-1747.ep12515868

[46] J. Riesz , J. Gilmore , P. Meredith , Biophys. J. 2006, 90, 4137–4144. 16565050 10.1529/biophysj.105.075713 PMC1459487

[47] R. Berera , R. van Grondelle , J. T. M. Kennis , Photosynth. Res. 2009, 101, 105–118. 19578970 10.1007/s11120-009-9454-y PMC2744833

[48a] G. Cerullo , C. Manzoni , L. Lüer , D. Polli , Photochem. Photobiol. Sci. 2007, 6, 135–144; 17277836 10.1039/b606949e

[48b] R. Borrego-Varillas , L. Ganzer , G. Cerullo , C. Manzoni , Appl. Sci. 2018, 8, 989.

[49] M. R. Chedekel , P. P. Agin , R. M. Sayre , Photochem. Photobiol. 1980, 31, 553–555. 10.1111/j.1751-1097.1980.tb02553.x 7384229

[50a] T. Ye , J. D. Simon , J. Phys. Chem. B. 2002, 106, 6133–6135;

[50b] T. Ye , J. D. Simon , J. Phys. Chem. B. 2003, 107, 11240–11244;

[50c] T. Ye , J. D. Simon , Photochem. Photobiol. 2003, 77, 41–45; 12856881 10.1562/0031-8655(2003)077<0041:tasfgo>2.0.co;2

[50d] Y. Tong , H. Lian , G. Jacob , P. Anna , S. E. Glenn , J. N. Robert , S. Tadeusz , D. S. John , Photochem. Photobiol. 2006, 82, 733–737;16542109

[50e] I. R. Piletic , T. E. Matthews , W. S. Warren , J. Phys. Chem. A. 2010, 114, 11483–11491; 20882951 10.1021/jp103608d PMC3334281

[50f] M. J. Simpson , J. W. Wilson , F. E. Robles , C. P. Dall , K. Glass , J. D. Simon , W. S. Warren , J. Phys. Chem. A. 2014, 118, 993–1003; 24446774 10.1021/jp4107475 PMC3983346

[50g] A. Brunetti , M. Arciuli , L. Triggiani , F. Sallustio , A. Gallone , R. Tommasi , Eur. Biophys. J. 2019, 48, 153–160. 30635668 10.1007/s00249-018-1342-y

[51] S. Premi , S. Wallisch , C. M. Mano , A. B. Weiner , A. Bacchiocchi , K. Wakamatsu , E. J. H. Bechara , R. Halaban , T. Douki , D. E. Brash , Science 2015, 347, 842–847. 25700512 10.1126/science.1256022 PMC4432913

[52a] C. Grieco , J. M. Empey , F. R. Kohl , B. Kohler , Faraday Discuss. 2019, 216, 520–537; 31012874 10.1039/c8fd00231b

[52b] C. Grieco , F. R. Kohl , Y. Zhang , S. Natarajan , L. Blancafort , B. Kohler , Photochem. Photobiol. 2019, 95, 163–175. 30317633 10.1111/php.13035

[53] C. Grieco , F. R. Kohl , A. T. Hanes , B. Kohler , Nat. Commun. 2020, 11, 4569. 32917892 10.1038/s41467-020-18393-w PMC7486937

[54a] I. L. Medintz , M. H. Stewart , S. A. Trammell , K. Susumu , J. B. Delehanty , B. C. Mei , J. S. Melinger , J. B. Blanco-Canosa , P. E. Dawson , H. Mattoussi , Nat. Mater. 2010, 9, 676–684; 20651808 10.1038/nmat2811

[54b] X. Ji , G. Palui , T. Avellini , H. B. Na , C. Yi , K. L. Knappenberger , H. Mattoussi , J. Am. Chem. Soc. 2012, 134, 6006–6017; 22394283 10.1021/ja300724x

[54c] D. Fan , C. Wu , K. Wang , X. Gu , Y. Liu , E. Wang , Chem. Commun. 2016, 52, 406–409. 10.1039/c5cc06754e 26526224

[55a] C. Du , X. Wu , M. He , Y. Zhang , R. Zhang , C.-M. Dong , J. Mater. Chem. B 2021, 9, 1478–1490; 33427844 10.1039/d0tb02659j

[55b] P. Yang , F. Zhu , Z. Zhang , Y. Cheng , Z. Wang , Y. Li , Chem. Soc. Rev. 2021, 50, 8319–8343; 34100489 10.1039/d1cs00374g

[55c] D. Wu , J. Zhou , M. N. Creyer , W. Yim , Z. Chen , P. B. Messersmith , J. V. Jokerst , Chem. Soc. Rev. 2021, 50, 4432–4483. 33595004 10.1039/d0cs00908c PMC8106539

[56] A. Corani , A. Huijser , T. Gustavsson , D. Markovitsi , P.-Å. Malmqvist , A. Pezzella , M. D'Ischia , V. Sundström , J. Am. Chem. Soc. 2014, 136, 11626–11635. 25078723 10.1021/ja501499q

[57] J.-H. Lin , C.-J. Yu , Y.-C. Yang , W. L. Tseng , Phys. Chem. Chem. Phys. 2015, 17, 15124–15130. 25820836 10.1039/c5cp00932d

[58] M. Chen , Q. Wen , F. Gu , J. Gao , C. C. Zhang , Q. Wang , Chem. Eng. J. 2018, 342, 331–338.

[59] H. Yin , K. Zhang , L. Wang , K. Zhou , J. Zeng , D. Gao , Z. Xia , Q. Fu , Nanoscale 2018, 10, 18064–18073. 30229779 10.1039/c8nr05878d

[60] B. Liu , X. Han , J. Liu , Nanoscale 2016, 8, 13620–13626. 27364882 10.1039/c6nr02584f

[61] X.-J. Kong , S. Wu , T.-T. Chen , R.-Q. Yu , X. Chu , Nanoscale 2016, 8, 15604–15610. 27511888 10.1039/c6nr04777g

[62] S. Quignard , M. D′Ischia , Y. Chen , J. Fattaccioli , ChemPlusChem 2014, 79, 1254–1257.

[63] A. Mavridi-Printezi , A. Menichetti , M. Guernelli , M. Montalti , Nanoscale 2021, 13, 9147–9159. 33978040 10.1039/d1nr01401c

[64] K.-Y. Ju , S. Degan , M. C. Fischer , K. C. Zhou , X. Jia , J. Yu , W. S. Warren , J. Biomed. Opt. 2019, 24, 1–13. 10.1117/1.JBO.24.5.051414 PMC6460485 30977334

[65] V. Balzani , A. Credi , F. M. Raymo , J. F. Stoddart , Angew. Chem. Int. Ed. 2000, 39, 3348–3391; 10.1002/1521-3773(20001002)39:19<3348::aid-anie3348>3.0.co;2-x 11091368

